# Marine Hospital at Cleveland

**Published:** 1845-06-01

**Authors:** 


MARINE HOSPITAL AT CLEVELAND.—We learn that an appropriation of $25,000 was made at the last session of Congress for the erection of a Marine Hospital at Cleveland. A board of Medical Officers of the Army were detailed some eight or ten years since, to determine the most suitable points of location for Marine Hospitals on the Western waters. They designated Cleveland on Lake Erie as one point, and a site was subsequently purchased by the United States for this object. We have no objections to our neighbors at Cleveland having a Marine Hospital, and we are glad that Congress has seen fit to indicate a sense of what is due to the Sailors navigating these Lakes, by some definite action on the subject. But we think that the necessity of a Hospital at Buffalo will not be diminished by the establishment of one at Cleveland, and we hope that the profession and citizens of this city will use efforts to procure it without further delay. Cleveland was doubtless selected by the board, as an accessible and convenient port, intermediate between Buffalo and Detroit, at a time when it was supposed one hospital would answer all the marine wants of Lake Erie. The great increase of Lake commerce, however, which has since taken place, and its prospective magnitude, require that hospital privileges should be provided at more than one point. In addition to that at Cleveland, an appropriation should be made forthwith for one at Buffalo, and probably, also, ere long, for one at Detroit.

Since the preceding was written we have seen by the newspapers, that an agent has been appointed to superintend the erection of the Hospital at Cleveland, and that the work will be immediately commenced.

